# Draft Genome Sequencing of *Methanobrevibacter oralis* Strain JMR01, Isolated from the Human Intestinal Microbiota

**DOI:** 10.1128/genomeA.00073-14

**Published:** 2014-02-20

**Authors:** Saber Khelaifia, Marc Garibal, Catherine Robert, Didier Raoult, Michel Drancourt

**Affiliations:** Aix Marseille Université, URMITE, UMR63 CNRS 7278, IRD 198, Inserm 1095, Marseille, France

## Abstract

*Methanobrevibacter oralis*, an anaerobic methanogenic archaeon, has been previously isolated from the human oral cavity. Here, sequencing a stool isolate (strain JMR01) yielded a 2.065-Mb genome with a 27.78% G+C content containing a total of 2,042 open reading frames and 3 clusters of regularly interspaced short palindromic repeat (CRISPR) loci with associated Cas proteins.

## GENOME ANNOUNCEMENT

*Methanobrevibacter oralis* was initially isolated from human subgingival plaque (1), and further studies associated this methanogen with subgingival diseases, including periodontitis (2). We recently demonstrated a significant correlation between the *M. oralis* charge and the severity of periodontitis (3). Furthermore, *M. oralis* DNA was detected in the human gut, and we previously reported the first isolation of *M. oralis* (strain JMR01) from the human gut (4). *M. oralis* strain JMR01 is a strict anaerobic archaeon with optimal growth at 37°C (pH 7.5) and 1.5 g/liter NaCl. Methane is produced by reducing acetate or H_2_-CO_2_ as an electron donor. Rumen fluid and vitamin solutions are required for growth and a volatile fatty acid mixture stimulates growth. Phylogenetic classification based on the 16S rRNA gene sequence (GenBank accession number KC616346) confirmed the affiliation of the *M. oralis* strain JMR01 herein sequenced with the genus *Methanobrevibacter* and yielded a sequence similarity of 99% with the reference strain *M. oralis* DSM 7256 (GenBank accession number HE654003).

The *M. oralis* strain JMR01 complete genome was sequenced by combining shotgun and 3-kb paired-end libraries using high-throughput 454 pyrosequencing (454 Life Sciences-Roche, Boulogne-Billancourt, France). Sequence reads were assembled using a Newbler assembler 2.8 (20120726_1306) (Roche), and 136 contigs were generated into 14 scaffolds. A preliminary open reading frame (ORF) prediction was conducted by automated annotation with Glimmer (http://www.cbcb.umd.edu/software/glimmer/) and RAST (5). The annotation was manually curated using BLAST and the nr database of NCBI. The CRISPR finder (http://crispr.u-psud.fr/Server/) was used to detect and identify CRISPR repeat and spacer sequences in the genome.

The *M. oralis* genome consists of a 2,065,764-bp circular chromosome (with a GC content of 27.78%). A total of 2,042 ORFs were recovered, encoding proteins involved in functions previously assigned in other related *Methanobrevibacter* organisms (6). Likewise, the *M. oralis* genome contains three CRISPR loci and associated proteins (Cas), as previously found in *Methanobrevibacter arboriphilicus* (7).

We previously observed that *M. oralis*, an inhabitant of the oral cavity, survives in the acidity of the stomach (S. Khelaifia and M. Drancourt, unpublished data), giving it an opportunity to establish itself in the digestive tract. Accordingly, we isolated *M. oralis* from the human gut. The availability of the *M. oralis* genome will give the opportunity to develop genotyping methods to investigate the diversity of this frequently detected human archaeon. Moreover, the isolation of a new *M. oralis* strain from the human gut, as well as its genome sequencing, can increase knowledge of this neglected component of the human gut microbiota.

### Nucleotide sequence accession numbers.

The *Methanobrevibacter oralis* strain JMR01 genome sequence has been deposited in EMBL under the accession numbers CBWS010000001 through CBWS010000060. The whole-genome shotgun project has been deposited in GenBank under the accession number CBWS000000000.
